# Cytokine Storm in COVID-19: Insight into Pathological Mechanisms and Therapeutic Benefits of Chinese Herbal Medicines

**DOI:** 10.3390/medicines11070014

**Published:** 2024-07-18

**Authors:** Qingyuan Yu, Xian Zhou, Rotina Kapini, Anthony Arsecularatne, Wenting Song, Chunguang Li, Yang Liu, Junguo Ren, Gerald Münch, Jianxun Liu, Dennis Chang

**Affiliations:** 1 Beijing Key Laboratory of Pharmacology of Chinese Materia Region, Institute of Basic Medical Sciences of Xiyuan Hospital, China Academy of Chinese Medical Sciences, Beijing 100091, China; 13693157577@163.com (Q.Y.); wenting_song1@163.com (W.S.); reek2003@163.com (J.R.); 2 Xiyuan Clinical Medical College, Beijing University of Chinese Medicine, Beijing 100029, China; 3 NICM Health Research Institute, Western Sydney University, Westmead, NSW 2145, Australia; p.zhou@westernsydney.edu.au (X.Z.); 20549389@student.westernsydney.edu.au (R.K.); 20205690@student.westernsydney.edu.au (A.A.); c.li@westernsydney.edu.au (C.L.); vickyliu9696@gmail.com (Y.L.); g.muench@westernsydney.edu.au (G.M.); 4 School of Science, Western Sydney University, Campbelltown, NSW 2560, Australia; 5 Pharmacology Unit, School of Medicine, Western Sydney University, Campbelltown, NSW 2560, Australia

**Keywords:** cytokine storm, COVID-19, acute respiratory distress syndrome, integrative Chinese medicines, curcumin

## Abstract

Cytokine storm (CS) is the main driver of SARS-CoV-2-induced acute respiratory distress syndrome (ARDS) in severe coronavirus disease-19 (COVID-19). The pathological mechanisms of CS are quite complex and involve multiple critical molecular targets that turn self-limited and mild COVID-19 into a severe and life-threatening concern. At present, vaccines are strongly recommended as safe and effective treatments for preventing serious illness or death from COVID-19. However, effective treatment options are still lacking for people who are at the most risk or hospitalized with severe disease. Chinese herbal medicines have been shown to improve the clinical outcomes of mild to severe COVID-19 as an adjunct therapy, particular preventing the development of mild to severe ARDS. This review illustrates in detail the pathogenesis of CS-involved ARDS and its associated key molecular targets, cytokines and signalling pathways. The therapeutic targets were identified particularly in relation to the turning points of the development of COVID-19, from mild symptoms to severe ARDS. Preclinical and clinical studies were reviewed for the effects of Chinese herbal medicines together with conventional therapies in reducing ARDS symptoms and addressing critical therapeutic targets associated with CS. Multiple herbal formulations, herbal extracts and single bioactive phytochemicals with or without conventional therapies demonstrated strong anti-CS effects through multiple mechanisms. However, evidence from larger, well-designed clinical trials is lacking and their detailed mechanisms of action are yet to be well elucidated. More research is warranted to further evaluate the therapeutic value of Chinese herbal medicine for CS in COVID-19-induced ARDS.

## 1. Introduction

Cytokine storm (CS) is a critical pathological process that describes the excessive release of cytokines induced by a noxious stressor, either infectious or non-infectious. Even though the initial release of cytokines is defensive, the dysregulated inflammation can cause a massive inflammatory cascade, leading to an irreversible end-organ dysfunction and even death [1]. The term “cytokine storm” was first proposed in 1993, characterised by the uncontrollable inflammatory states in graft versus host disease [2]. Now it has been frequently used in lay media to describe a situation in which inflammatory cytokines are overly secreted in response to certain diseases. These include coronavirus disease-19 (COVID-19) acute respiratory distress syndrome (ARDS), caused by severe acute respiratory syndrome coronavirus 2 (SARS-CoV-2) infection.

Clinical evidence demonstrates that the invasion of SARS-CoV-2 can disrupt immune activation, leading to CS and eventually ARDS [3,4], which has been identified as a key factor in COVID-19 mortality. Clinically, COVID-19 ARDS is diagnosed when a patient with a confirmed SARS-CoV-2 infection develops signs and symptoms meeting the Berlin ARDS criteria [5]: 1. the ratio of the partial pressure of oxygen in arterial blood (PaO_2_) to the fraction of the oxygen in the inspired air (FiO_2_) is less than 300; 2. there is acute onset; and 3. there are bilateral lung infiltrates on the chest radiography of a non-cardiac origin. COVID-19 ARDS typically results in impaired alveolar homeostasis, pulmonary fibrosis, endothelial inflammation, vascular thrombosis and immune cell activation leading to dyspnoea and hypoxia [6]. If left untreated, extra-pulmonary complications including acute kidney injury, hepatic injury, cardiomyopathy, encephalopathy, thrombosis and death can occur. Even after survival, the patient may experience various long-term sequelae of COVID-19 ARDS, known as post-acute COVID-19 syndrome (PACS), including fatigue, dyspnoea, chest pain, cognitive disturbances, arthralgia and an overall decline in quality of life [7].

In recent years, increasing attention has been drawn to CS due to the COVID-19 pandemic. Although it has been more than four years since the COVID-19 pandemic began, it is not easy to declare an end to the pandemic thus far. Since 2019, an increased amount of research has been devoted to elucidating the pathological mechanisms of ARDS associated with COVID-19 and the treatment strategies for hyper-inflammation induced by the CS, in an attempt to reduce severity and mortality. Whilst other coronaviruses have been studied to shed light on the pathology of COVID-19, especially early in the pandemic, it has been shown that coronaviruses, i.e., SARS-CoV, MERS-CoV and SARS-CoV-2, produce different profiles of immune cascades and cytokines [8]. The slightly varied pathological mechanisms are closely related to clinical prognosis and disease outcomes. Thus, the typical molecular basis of pathogenesis involved in SARS-CoV-2 remains to be explored for clinical prognosis and disease outcome that cannot be guided by SARS-CoV or MERS-CoV.

Despite the research devoted to elucidating the immunopathological mechanisms behind ARDS induced by SARS-CoV-2 infection, efficacious treatment of severe COVID-19 has yet to be discovered. The wide use of mRNA and other COVID-19 vaccines have prevented severe illness and death through development of immunity to the virus. However, vaccines alone have not been enough to bring the crisis under control in specific regions or circumstances [9]. Particularly for people who are at higher risk of severe illness or death, additional preventative and treatment strategies are still an integral part of combating COVID-19 [10,11]. Based on the available research and clinical observations, the recommended treatment strategies for COVID-19 ARDS are largely directed at controlling SARS-CoV-2 infection, ensuring hemodynamic stability, attenuating the corresponding CS and consequent multi-organ failure [12]. To attenuate CS, glucocorticoids in low doses have been considered to decrease the production of inflammatory mediators [13,14]. However, the onset and duration of glucocorticoid treatment presents an issue, as early intervention is likely to cause a higher viral load due to immunosuppressive effects and later intervention may induce a secondary infection. Collectively, the clinical benefit of glucocorticoids in COVID-19 treatment is based on the selection of the correct dose, selected patient and appropriate timing. Thus, glucocorticoid therapy as a first-line treatment for ARDS in COVID-19 is not recommended [15]. The blockage of key inflammatory mediators, through antibody-mediated neutralisation, have been considered for SARS-CoV-2-induced ARDS, such as tumour necrosis factor (TNF)-α inhibitors and interleukin (IL)-1 inhibitors [16]. However, their clinical efficacy remains disputed, in addition to the expense and adverse reactions of the treatment [17]. Thus, none of the current treatment strategies are satisfactory as most have yielded mixed results in clinical settings or mixed effectiveness in selected subgroups. Therefore, there remains an urgent need for in-depth understanding of the immunopathological mechanisms in ARDS induced by SARS-CoV-2 to continue the development of effective treatments. 

Recent preclinical and clinical evidence has shown a number of Chinese herbal formulations exhibited potential therapeutic benefits in the management of ARDS as add-on treatments, especially for attenuating clinical symptoms, and reduce the severity and mortality of COVID-19 [18,19]. The mechanisms behind this phenomenon are complicated, in part due to the composition and the variety of Chinese herbal formulations, which have demonstrated anti-inflammatory, antiviral and immunomodulatory effects [20,21]. The multi-component and multi-target approach embedded in the composition of herbal formulations may support the treatment strategy to help address the complex pathogenesis in ARDS. However, the evidence for the use of Chinese herbal formulations in SARS-CoV-2 infection-related CS exists largely in studies that are not randomised controlled trials, which is essential to rectify. This review aims to provide a comprehensive overview of the pathological mechanisms of CS in COVID-19 ARDS and key therapeutic targets based on the current available studies from 2019 to 2024 and the therapeutic benefits of Chinese herbal medicines as a promising adjunct therapy in the treatment of the disease.

## 2. The Pathology of CS in SARS-CoV-2 Infection-Induced ARDS

COVID-19 is a complicated disease process, differentially affecting patients with increased age and comorbidities [22]. Despite the incomplete knowledge of the immunopathological mechanism, we are gaining better understanding of the complex pathophysiology of severe COVID-19, including impaired alveolar epithelial cell regeneration, myeloid immune cell infection and pathological pulmonary fibrosis [23]. These new developments in the understanding of SARS-CoV-2 can potentially lead to the development of a safe and effective treatment for ARDS, alongside continued preventative efforts through vaccination.

### 2.1. SARS-CoV-2 Infection and Mild COVID-19

Like most human coronaviruses, SARS-CoV-2 affects the respiratory system [24], infecting the ciliated cells in the upper respiratory tract epithelium, primarily in the nasopharynx and trachea. Nasal pseudostratified ciliated epithelial cells have been identified as a primary target [25,26,27]. Entry into host cells is regulated through the interaction of the spike glycoprotein and the extracellular receptor binding domain of ACE2 [28,29]. Upon entry, the viral genome directly initiates the production of viral proteins through modified endoplasmic reticulum networks to form a viral replication complex [30]. The modified endoplasmic reticulum contains double-stranded RNA (dsRNA), which can be detected by cytoplasmic pattern recognition receptors such as toll-like receptor 3 (TLR3) and TLR7, and initiates an acute inflammatory response promoting the production of type I and III interferons [31,32]. The activated type I and III interferon (IFN) responses act as the first line of defence against viral infection. The inflammatory response, involving the translocation of nuclear factor-κB (NF-κB), leads to the release of major proinflammatory cytokines such as interleukin (IL)-1, IL-6 and TNF-α. Adjacent cells may release further interferons, chemokines and cytokines, in response to the inflammatory cytokine release through the Janus kinase–signal transducer and activators of transcription (JAK-STAT) signalling pathway, which stimulates the development of adaptive B and T cell responses to clear the virus. Ultimately, the viral infection results in the induction of an immune response that leads to an adjunct inflammatory response. The inflammatory, immune or combination of responses lead to the symptoms that are experienced in mild or self-limiting COVID-19, which includes cough, fever, headache, myalgia and diarrhea during the inflammatory response [33].

### 2.2. The Role of CS in Severe COVID-19 and the Development of COVID-19 ARDS

Severe COVID-19 generally differs from mild or self-resolving COVID-19 by the extent of viral spread, which typically proceeds down the trachea and the bronchoalveolar tree. Presently, a delayed or poor interferon response is believed to be the causative mechanism in the continued spread of SARS-CoV-2 to the lower respiratory tract [34]. The dysregulated host IFN responses have been known to be associated with the progression of severe symptoms of COVID-19 [32]. Low induction of local and systemic interferon responses has been observed in patients with severe COVID-19, whilst patients with autoantibodies to IFN and patients with abnormal interferon signalling also have a predisposition for severe COVID-19 [8,35]. The delayed or minimal interferon response facilitates the virus infecting alveolar epithelial cells, where viral replication and reactive oxygen species (ROS) production result in alveolar cell damage and death. The loss of alveolar type 1 and 2 cells contribute to the disruption of the alveolar epithelium, initiating a cascading inflammatory response involving cytokines and chemokines such as IL-1, IL-6 and TNF-α. In nearby alveolar epithelial cells, the pro-inflammatory cytokines may activate the JAK-STAT pathway or result in NF-κB nucleus translocation, leading to the escalated production of inflammatory cytokines. In addition, alveolar type 2 (AT2) cells will proliferate in an attempt to repair damaged pulmonary tissue, but adopt a cell state termed damage-associated transient progenitor (DATP) due to the increased inflammatory state [36]. AT2 cells in the DATP state are incapable of differentiating into alveolar type 1 (AT1) cells, sustaining the hypoxic state of the patient due to the inadequate restoration of damaged or dead AT1 cells [36]. 

Individual or combinations of factors such as hypoxia, cytokines and chemokines can result in a leaky state where the endothelium and epithelium are loosely bound together. This will lead to the activation of the endothelium, resulting in fluid leakage, exposure of the subendothelial extracellular matrix (ECM) and coagulation factor release [34]. Upon subendothelial ECM exposure, platelets are activated and aggregate to seal the damage, but release pro-coagulative factors. In addition to coagulation factors released as part of a physiological inflammatory response, migrating immune cells also contribute to increased platelet activation and aggregation, leading to increased clotting. The circulating immune cells that migrate typically express and release tissue factor, which increases fibrin production through the extrinsic pathway of coagulation. Fibrin is a major component of thrombi and the hyaline membranes responsible for the pathological coagulation and pulmonary fibrosis observed in lethal COVID-19 ARDS [23]. Monocytes and neutrophils express high levels of tissue factor [37,38], which activates factor X. Activated factor X produces thrombin which leads to the cleavage of fibrinogen to fibrin through the extrinsic pathway of coagulation. Additionally, the activated neutrophils form neutrophil extracellular traps (NETs), which increase coagulation through the intrinsic pathway, by their direct activation of factor XII [39,40]. All these factors induce a major imbalance between thrombosis and thrombolysis in addition to the immune and inflammatory response to SARS-CoV-2 infection, leading to the development of thrombi and hyaline membranes in the lungs. 

The activated endothelium, proinflammatory mediators released by damaged or dead cells and resident immune cell cytokine and chemokine release results in the large migration of circulating white blood cells, as well as a progressive increase in the inflammatory mediators in the tissue. During the initial stages of the SARS-CoV-2 infection, a large neutrophil response occurs for defensive purposes, but as the infection progresses and further lung damage occurs, the neutrophil response becomes pathological. The pathological neutrophils express proteins involved in inflammatory pathways, degranulation and NETs [39,41] which contributes to the lung injury seen in COVID-19 ARDS [42,43]. The macrophages and monocytes that migrate to the lung can be infected by SARS-CoV-2, creating a positive feedback cycle that leads to further inflammation [44,45]. Infection of macrophages and monocytes induces pyroptosis, a type of inflammatory cell death, through the NACHT, LRR and PYD domains containing protein 3 (NLRP3) inflammasome, neutralising the virus but releasing large amounts of IL-1β and IL-18 in the process. Another important factor is the activation of the complement system, through the alternative or lectin pathway [46,47], which drives the formation of NETs [38,39] and migration of CD16+ cytotoxic T cells [48]. These T cells promoted microvascular endothelial cell damage and the release of chemokines like IL-8 and chemokine ligand 2 (CCL-2), attracting circulating immune cells to the lungs and continuing the positive feedback cycle [34,48]. 

Ultimately, the combination of alveolar epithelial damage, migrating myeloid immune cell infection and subsequent pyroptotic death as well as the release of cytotoxic proteins by pathological neutrophil and CD16+ T cell degranulation leads to lung hyperinflammation. This hyperinflammation is characterised by elevated circulating cytokines, but especially epithelial-derived IL-6 and macrophage or monocyte-derived IL-1β, which differentiates COVID-19 pulmonary disease from other viral and bacterial-induced pneumonias [23]. This drives further alveolar activation and death to other areas of the lung, sustaining the hyperinflammation through a positive feedback cycle. Overall, the increased inflammation, exudate and hyaline membranes impair the alveolar function and regeneration in the lungs, leading to a decrease in the oxygen exchanging capability of the lung. Sustained loss of oxygen exchanging capability results in a hypoxemic state that defines ARDS, leading to multiple organ dysfunction and death without rapid reversal.

**Figure 1:**
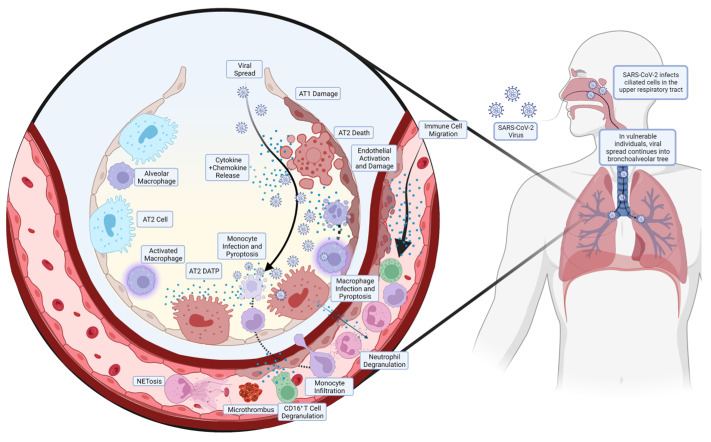
Pathogenesis of COVID-19 ARDS. The SARS-CoV-2 enters the upper respiratory tract and infects the ciliated cells in the area, predominantly in the nasopharynx. In most individuals, the virus is cleared at this stage by the corresponding inflammatory and immune response, but in some individuals with diminished or absent responses, the virus continues to spread. The SARS-CoV-2 will spread down the bronchoalveolar tree, ultimately ending up in the alveoli where it infects alveolar type 2 (AT2) cells. This results in the release of cytokines and chemokines, activating and disrupting the endothelial layer, which release more chemoattractant, leading to the migration of circulating immune cells. Monocytes, in addition to resident alveolar macrophages, migrate to the site of infection but become infected and undergo pyroptosis, releasing large amounts of pro-inflammatory cytokines. Neutrophils create neutrophil extracellular traps (NETs), in a process known as NETosis, ultimately creating microthrombi. In addition, neutrophils and CD16+ T cells release pro-inflammatory proteins, contributing to the pulmonary hyperinflammation that precedes ARDS.

A summarised diagram of the pathogenesis of COVID-19 ARDS is shown in Figure 1. 

### 2.3. Critical Signalling Pathways, Transcription Factors and Their Downstream Products as Potential Therapeutic Targets for ARDS in Severe COVID-19 Induced by CS

#### 2.3.1. JAK-STAT Pathway

The JAK-STAT pathway is a critical pathway that extracellular cytokines, IFNs and growth factors utilise to control cellular function through gene expression. The pathway provides a direct connection for membrane receptors to the nucleus, thereby allowing control of gene expression and thus cellular function. When a cytokine or other ligand activates the cellular receptor, JAKs become activated and phosphorylate other JAKs and the receptor, allowing STATs to dock and bind to the cytoplasmic domain of the receptor. The STATs are subsequently activated, allowing the dimerization of STATs, which translocate to the nucleus. Once inside the nucleus, it can directly bind to genetic material and regulate cellular gene expression and thus cellular function [49].

**Figure 2:**
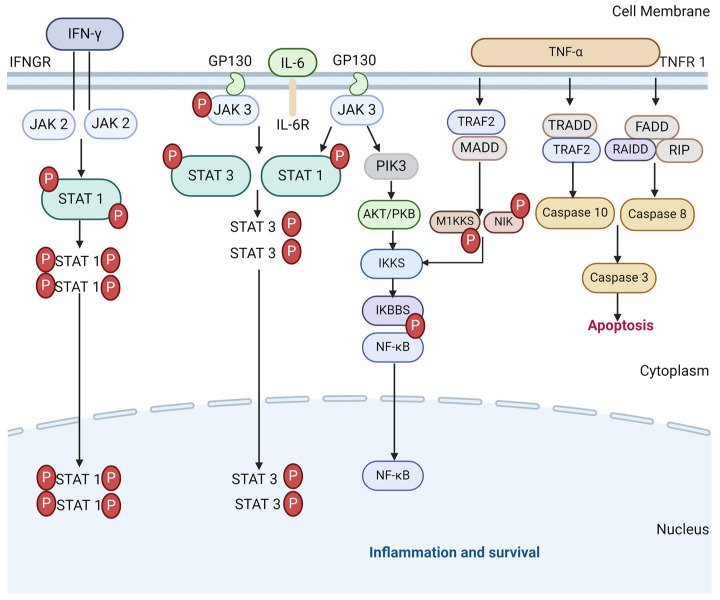
The JAK-STAT signalling pathways in CS and cell survival. TNF-α binds to TNF receptor 1 to initiate the downstream of protein molecules. The TNFR1–associated death domain protein (TRADD) interacts with TRAF 2 to activate caspase 10 and 3 and lead to apoptosis. Fas-associated death domain (FADD) interacts with RAIDD protein to activate caspase 8 that also activates 3 (and 6 and 7 in some cases) to induce apoptosis. TRAF 2 and TRADD also activate IKK phosphorylation to produce NF-κB dimers via M1KKS and NIK. IL-6 similarly activates NF-κB dimers as with the JAK/STAT3 pathway to express inflammatory genes. IFN-γ follow suit by binding to IFNGR to transduce JAK2/STAT1 pathway to contribute to the initiation of SIRs via pro-inflammatory cytokines.

The JAK-STAT pathway plays a critical part in the development of ARDS in severe COVID-19, as a driver of the physiological immune and inflammatory response to the viral infection. However, it also sustains the pathological release of pro-inflammatory cytokines leading to hyperinflammation. The pro-inflammatory cytokines that signal via the JAK-STAT signalling pathway include IL-2, IL-6, IL-8 and TNF-α, all of which are elevated in severe COVID-19 [50,51], as well as antiviral or anti-inflammatory cytokines such as IFN-γ and IL10, which are also elevated in severe COVID-19 [51,52]. Therefore, they hold the potential to cut off pathological reactions in COVID-19 [53]. As such, JAK inhibitors have been discussed in many studies as a potential therapeutic agent to suppress hyperinflammation and the activities of pro-inflammatory cytokines [53,54,55,56,57,58]. A meta-analysis suggested that several JAK inhibitors exerted a benefit for risk reduction concerning major COVID-19 outcomes when added to the standard of care in patients [59]. However, challenges remain regarding the precise timing of treatment in future trials, along with the control of viral replication by associating antiviral molecules under the immune-suppressive condition. A diagram of the JAK-STAT pathway in inflammation and survival is shown in Figure 2. 

#### 2.3.2. NF-κB Signalling Pathway

NF-κB is a central regulatory transcription factor which is responsible for mediating inflammatory responses through its effects on innate and adaptive immune systems. The transcription factor induces the expression of pro-inflammatory genes, leading to the release of cytokines and chemokines, and regulates the survival and function of innate immune cells and T cells. Activation of NF-κB can be through the canonical pathway and alternative pathway, which differ in their response stimuli to the process by which NF-κB translocates into the nucleus of the cell. Canonical activation of NF-κB occurs when a cytokine, pattern recognition, TNF or other receptor binds to its ligand. This induces ubiquitin-dependent degradation of IκBα by an IκB kinase (IKK) complex, leading to NF-κB translocation in the nucleus [60]. Alternative or non-canonical activation of NF-κB responds to a smaller set of stimuli and involves p100 ubiquitination by NF-κB inducing kinase, which leads to the generation of NF-κB2 p52 and nuclear translocation of this non-canonical NF-κB complex [60]. Currently, it is believed that the canonical pathway is involved in all aspects of immune response, whilst the non-canonical pathway is believed to only be involved in the adaptive immune system.

As with the JAK-STAT pathway, the NF-κB transcription factor is a critical part of the initial physiological and later pathological immune and hyperinflammatory response to the infection by the SARS-CoV-2. The NF-κB transcription factor is activated by cytokines such as IL-1β, IL-6 and TNF-α, which are elevated in severe COVID-19 [51]. As such, the inhibition of NF-κB would allow the targeting of multiple cytokines/chemokines which are expected to have much higher therapeutic potential than a single cytokine target to prevent the further cascade, especially for the critical stage COVID-19 patients, without the inhibition of antiviral cytokines such as IFN-γ [61].

#### 2.3.3. Mitogen-Activated Protein Kinase Signalling Pathway

The mitogen-activated protein kinase (MAPK) family refers to similar intracellular signalling pathways that coordinate cellular responses to various stimuli, maintaining communication from the cell membrane to the nucleus. The MAPK family can be further categorised into typical and atypical MAPK signalling cascades, with typical cascades (composed of three kinases) being phosphorylated in succession [62]. Typical MAPK signalling cascades follow the hierarchy of a MAPK kinase (MAPKK) phosphorylating a MAPK kinase (MAPKK), which then phosphorylates a MAPK. Examples include the extracellular signal-regulated kinases protein homologs 1 and 2 (ERK 1/2), the c-Jun N-Terminal Kinase 1, 2 and 3 (JNK 1/2/3) and the extracellular signal-regulated kinase 6 (p38) [63]. The ERK, JNK and p38 MAPKs play a vital role in cellular functions such as proliferation, development and apoptosis [64]. They also play a critical role in the innate immune response to pathogens, through stimulation of pro-inflammatory cytokines in response to the activation of oxidative, TLRs and tissue necrosis factor receptors [64,65]. 

Similar to the JAK-STAT and NF-κB signalling pathways, MAPK signalling pathways are a critical part of the initiation of the innate immune response to SARS-CoV-2 infection. Whilst this response is initially physiological, the sustained release of pro-inflammatory cytokines leads to the development of COVID-19-induced ARDS. Specifically, IL-1β, TNF-α and IL-6 [51], all of which are elevated in COVID-19, are potent activators of the p38 MAPK signalling cascade, which itself leads to further production of these cytokines [66]. Given the significant impact of these cytokines on the development of CS in SARS-CoV-2 infection, inhibition of p38 MAPK signalling has been identified as a possible therapy to suppress this uncontrolled inflammation [67,68].

#### 2.3.4. NLRP3 Inflammasome

The NLRP3 inflammasome is a complex that is largely responsible for cellular defences against viruses, as it induces pyroptosis, as well as cytokine IL-1β and IL-18 secretion. Priming of the NLRP3 inflammasome occurs in response to pathogen-associated molecular pattern (PAMP) molecules and damage-associated molecular patterns (DAMPs), IL-1β or TNF leading to canonical activation of the NF-κB transcription factor. As NF-κB nucleus translocation occurs, pro-IL-1β and pro-IL-18 are formed, in addition to caspase-1 and inactivated NLRP3. Activation occurs in response to oxidative stress, DAMPs, viral invasion, ionic influx or efflux, leading to the formation of the NLRP3 inflammasome, then leading to the activation of caspase-1, which catalyses the formation of functional IL-1β and IL-18 as well as activated gasdermin D. Gasdermin D induces pore formation in the membrane, leading to the release of higher productions of cytoplasmic constituents including IL-1β and IL-18 into the extracellular environment, and results in increased cell death.

**Figure 3:**
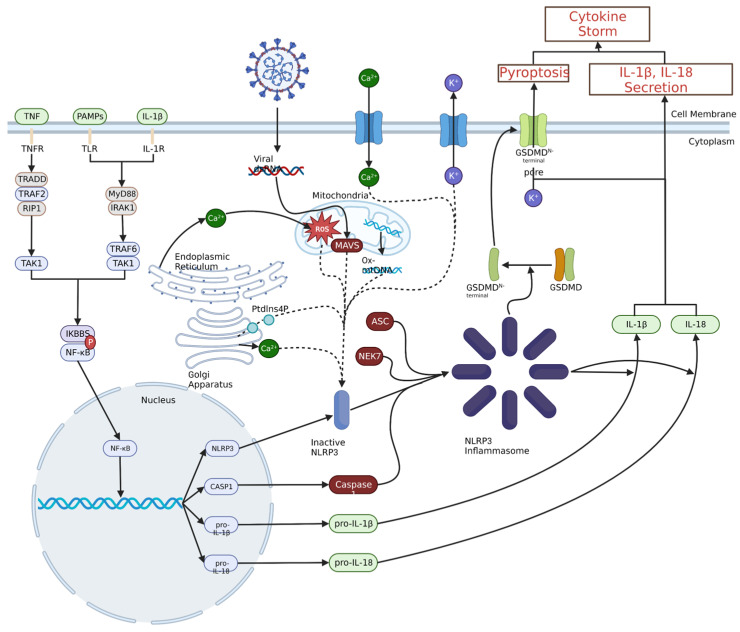
Priming and activation of the NLRP3 inflammasome. Priming of the NLRP3 inflammasome typically results from pathogen- or damage-associated molecular pattern interaction with toll-like receptors (TLRs), IL-1β binding to IL-1 receptor or TNF binding to TNF receptor. This ultimately leads to NF-κB nuclear translocation through a signalling cascade, which leads to the production of NLRP3, caspase 1 (CASP1), pro-IL-1β and pro-IL-18. A variety of intracellular and extracellular events can activate the NLRP3 inflammasome, including viral invasion, Ca^2+^ influx or K^+^ efflux, ROS, or oxidised mitochondrial DNA. These stimuli increase the amount of inactive NLRP3, which can then oligomerize with ASC, NEK7 and CASP1 to form the NLRP3 inflammasome. The NLRP3 inflammasome can then convert pro-IL-1β and pro-IL-18 to IL-1β and IL-18 and catalyse the formation of the gasdermin D N-terminal pores, leading to the release of pro-inflammatory cytokines and initiation of pyroptosis, respectively.

As with JAK-STAT signalling and the NF-κB transcription pathway, the NLRP3 inflammasome is a pertinent part of the innate immune system response to the SARS-CoV-2 infection, but later in the infection, it leads to the development of and sustains the hyperinflammation in the lungs. Therefore, inhibition of the NLRP3 inflammasome could potentially lead to a corresponding decrease in the levels of strongly pro-inflammatory cytokines, especially IL-1β, which is released from pyroptotic macrophages and monocytes in large amounts in COVID-19 ARDS [23,51,69,70]. A diagram of NLRP3 inflammasome and NF-κB signalling in a large secretion of IL-1β and IL-18 is shown in Figure 3.

## 3. Chinese Herbal Medicines as Potential Therapeutic Agents for CS in Relation to ARDS

### 3.1. Integrative Chinese Herbal Medicines with a Holistic Approach

CS in COVID-19 ARDS is a complex disease process. The recent findings will shed light on the development of the treatment strategy, particularly revolving around the key therapeutic targets such as major cytokines (TNF-α, IL-1β), JAK-STAT, NF-κB and NLRP3 inflammasome signalling that hold the potential to cut off pathological reactions from mild to severe COVID-19 ARDS.

Chinese herbal medicines with a multi-component and multi-target approach provide an attractive alternative or adjunct therapy to single-entity, single-target pharmaceuticals for the prevention and treatment of complex conditions such as CS. The Chinese herbal medicines, when used in conjunction with conventional antiviral and anti-inflammatory drugs, provide better health outcomes in COVID-19 patients under high risk of developing severe consequences. Thus, Chinese herbal medicine may serve as an effective adjunctive therapy in combating COVID-19-related symptoms induced by inflammatory responses. In addition, many phytochemicals derived from Chinese herbal medicines exhibit broad anti-inflammatory and anti-cytokine activities which can be considered as candidates for future drug development to address the complex pathogenesis of CS. In this section, we review the current available research in Chinese herbal medicines, as herbal formulations, single herbal extracts or isolated compounds, for CS-associated COVID-19 ARDS.

### 3.2. Promising Chinese Herbal Medicines for ARDS with Preclinical and Clinical Evidence

#### 3.2.1. Xuebijing Injection

The Xuebijing (XBJ) injection is a patented formulation with *Carthamus tinctorius* L. (Honghua), *Paeonia lactiflora* Pall. (Chishao) and *Ligusticum chuanxiong* Hort. (Chuanxiong) as main ingredients. XBJ is widely used to treat sepsis, systemic inflammatory response syndrome and multiple organ dysfunction syndrome (MODS) in Chinese medicine [71]. The capacity of XBJ in mitigating CS and therefore ARDS in severe COVID-19 has been studied in several clinical and preclinical trials.

A meta-analysis was conducted to examine the efficacy and mechanism of action of the XBJ injection in ARDS based on 15 RCTs involving 2778 patients (13 ARDS and 2 severe pneumonia). Their findings support the use of XBJ in ARDS and severe pneumonia as evidenced by reduced mortality rates [risk ratio, 0.64 (95% credible interval (CrI), 0.54–0.77)], reduced ICU stay times [mean difference (MD), −4.51 (95% CrI, −4.97–−4.06)], reduced TNF-α [(MD), −1.23 (95% CrI, −1.38–−1.08)] and IL-6 [(MD), −1.15 (95% CrI, −1.52–−0.78)] levels [72]. The finding is in line with a more recent meta-analysis including four RCTs and three observational studies including 204 patients in the XBJ group and 183 patients in the routine treatment group [73].

A prospective randomised, controlled trial investigated the preventive effect of the XBL injection for 14 days (Tianjin Chase Sun Pharmaceutical Co., Tianjin, China; China lot number 1304291, 1401091 and, 1501261) against CS for 57 patients with severe COVID-19 [74]. The efficacy of the combined treatment of XBJ (50 mL diluted in 100 mL of normal saline, 60 min per injection for every 12 h) and standard care was compared with standard care alone (nutritional support, oxygen therapy, antiviral therapy with interferon-α inhalation, antibiotic agents and non-invasive and invasive ventilation, if necessary). Their research findings suggest that the integrative treatment significantly reduced the secretions of serum IL-6, TNF-α and IL-8 and was associated with a significant reduction in the need for mechanical ventilation, rate of septic shock, the proportion of critical illness, the duration of improvement of the main clinical symptoms and length of ICU stay. However, the trial presented a small sample size (*n* = 57).

Several animal studies have explored the capability of XBJ in reducing CS in septic models. Wang et al. found that XBJ (catalog number: z20040033, batch number: 1905061) significantly improved the survival of septic mice induced by cecal ligation and puncture (CLP). The reduced CS was manifested as decreased proinflammatory cytokines IL-6 and IL-1β in serum [75]. Another study suggested that the XBJ injection at 18 mL/kg stimulated Treg differentiation and moderately inhibited Th17 differentiation in vitro. It modulated the balance between proinflammation and anti-inflammation by reducing serum TNF-α and IL-6 and facilitating the expansion of IL-10 as well as Tregs. Furthermore, it prevented neutrophil infiltration into the lungs which may be linked with attenuated ARDS as observed in COVID-19 [76]. In addition, XBJ was found to reduce the production of TNF-α, IL-6 and IL-1β in the lung tissue in LPS-induced acute lung injury (ALI) mouse models [77]. A systematic review of 90 studies conducted over the last 20 years on the mechanisms of action of XBJ in clinical applications suggests that XBJ can reduce IL-2, IL-4, TNF-α and oxidative stress by modulating various signalling pathways, such as PI3K-AKT, NF-κB and MAPK. Additionally, it plays a role in cell protection, stabilizing haemodynamics, regulating metabolic processes and enhancing immune function [78].

#### 3.2.2. Huashi Baidu Formula

Huashi Baidu Formula (HSBD) was developed for the treatment of COVID-19 after the outbreak. HSBD is composed of 14 Chinese herbs, including *Ephedra sinica* Stapf (Mahuang), *Pogostemon cablin* (Blanco) Benth. (Huoxiang) and Hydrated calcium sulphate (Shigao) as the main ingredients [79]. The therapeutic effect of HSBD (China Academy of Chinese Medical Sciences) as an add-on therapy with Lopinavir-Ritonavir was investigated in the treatment of COVID-19 ARDS. 

An RCT suggested that an HSBD granule taken (10 g, twice daily) for 14 days, plus standard care, demonstrated significantly shorter recovery times for fever than that of the standard care alone in 204 patients with laboratory-confirmed COVID-19 with *n* = 102 per group. The disappearance rate of symptoms like cough, fatigue and chest discomfort was significantly greater in the treatment group [79]. More patients in the combined treatment group showed improvement in chest computed tomography (CT) imaging. However, there was no significant difference in the conversion time of the virus between the two groups, suggesting that the main target of the HSBD was related to calming CS rather than clearing the viral load. A non-randomised, controlled trial showed that 20 adult patients with laboratory-confirmed COVID-19 treated by HSBD (137 g twice daily, orally) with other traditional Chinese medicine injections (arm 3) had a shorter clinical remission time and significantly lowered levels of lymphocyte count and IL-6 compared to the group treated by Lopinavir-Ritonavir alone (arm 1, *n* = 20, 500 mg twice daily, orally) or Lopinavir-Ritonavir combined with the HSBD (arm 2, *n* = 20). However, the arm 3 treatment group involves multiple Chinese herbal formulations which make it difficult to assess the therapeutic value of the HSBD [80].

Preclinical studies and network pharmacology analysis were conducted to explore the mechanisms of the HSBD against CS in COVID-19. One analytical chemistry study has characterised and identified 216 compounds in the HSBD using ultra-high-performance liquid chromatography with quadrupole time-of-flight mass spectrometry (UPLC-QTOF-MS) [81]. Then, network pharmacology analysis of the HSBD and its main chemical compounds demonstrated its multi-component and multi-target action against severe COVID-19 and acute lung injury (ALI). Among all the identified compounds, baicalein and quercetin were the top two bioactive compounds of the HSBD which had high affinity with ACE2 [82], and other compounds such as emodin, aloe-emodin, rhein and luteolin acted on regulating inflammation, oxidative damage and apoptosis in an inflammatory environment by LPS stimulation [83]. Furthermore, Xu et al. (2023) characterised 343 chemical compounds in the HSBD using UPLC-QTOF-MS. In their in vitro tests screening for anti-inflammatory compounds, licochalcone B, glycyrrhisoflavone and echinatin demonstrated over 90% inhibition on IL-1β matured form expression in severe fever with thrombocytopenia syndrome virus (SFTSV)-induced THP-1 macrophage at 10 μM. These three compounds also reduced SARS-CoV-2-induced IL-1β P17 release in a dose-dependent manner. These results are not in line with the predicted anti-inflammatory compounds using network pharmacology. This study partly supports the mechanisms of action of the HSBD in combating SARS-CoV-2-induced inflammation by targeting IL-1β expression [84]. However, the possibility of relevance to the NLRP3 pathway was not explored in this study. Furthermore, the interactions of these bioactive compounds in the HSBD, and their relevant key signalling pathways such as NF-κB or JAK-STAT, has not been investigated. 

#### 3.2.3. Lianhua Qingwen Granules/Capsules

Lianhua Qingwen (LHQW) is a classic formulation used to treat influenza. LHQW is formulated with *Lonicera japonica* Thunb. (Jinyinhua) and *Forsythia suspensa* (Thunb.) Vahl (Lianqiao) as the main ingredients. 

The therapeutic value of LHQW as an adjunct therapy have been explored in multiple clinical trials. A multi-centred open-label RCT suggests that a 14-day treatment of LHQW (4 capsules, thrice daily, *n* = 410 patients, Shijiazhuang Yiling Pharmaceutical Co., Ltd., Shijiazhuang, China), plus routine treatment, resulted in higher recovery rates, improvements in chest CT imaging and a shorter median recovery time for symptoms such as fever, fatigue and coughing compared with routine treatment alone (oxygen therapy, antiviral medications and symptomatic treatments, *n* = 405 patients) [85]. Although no inflammatory markers were measured, the combined therapy has been shown to prevent the progression from mild to severe in COVID-19 ARDS. Another randomised, controlled, non-blinded research study with 292 suspected and diagnosed cases of COVID-19 showed that a 14-day treatment comprising a combination of LHQW (6 g per bag, 3 times a day), Huoxiang Zhengqi dropping pills (2.6 g in each bag, twice a day, Chinese medicine Z20000048, Tianjin Tasly Pharmaceutical Group Co., Ltd., Tianjin, China) and antiviral (oseltamivir, arbidol, ribavirin) and antimicrobial drugs (penicillins, cephalosporins, ofloxacin, macrolides, etc.) lowered the proportion of patients who progressed to severe disease and the utilisation rate of antibiotics compared with the group using antiviral and anti-microbial drugs alone [86]. 

The potential pharmacological action of LHQW in attenuating CS was explored in network pharmacology analysis and in vitro studies. A study by Li et al. (2020) investigated the antiviral and anti-inflammatory activity of LHQW against SARS-CoV-2 in vitro. Their results demonstrate that LHQW not only reduced the SARS-CoV-2 replication, but also inhibited pro-inflammatory cytokines (TNF-α, IL-6, CCL-2/MCP-1 and CXCL-10/IP-10) production at the mRNA levels [87]. However, the relevant signalling pathways were not explored. Two network pharmacology analyses illustrated that IL-6 receptor/IL-6/IL-6 receptor subunit beta were the main target of LHQW, and the main contributing bioactive compounds were quercetin, luteolin and wogonin [88,89]. 

#### 3.2.4. Qingfei Paidu Decoction

Qingfei Paidu Decoction (QFPD) was specifically designed by the State Administration of Traditional Chinese Medicine of China to treat mild to severe COVID-19 after the outbreak. The key ingredients of QFPD include *Ephedra sinica* Stapf (Mahuang), *Glycyrrhiza uralensis* Fish. (Zhigancao) and *Prunus armeniaca* L. var. *ansu* Maxim. (Kuxingren). It is recommended to be used with conventional treatments to increase hospital discharge rates, reduce severity and reduce critical cases. 

A meta-analysis based on fifteen trials (sourced from both English and Chinese databases) concludes that QFPD, used together with pharmaceutical medicines, significantly improved the cure rate and lung CTs of COVID-19 patients and reduced severity and death in COVID-19 patients [90]. The conclusion supports the combined use of QFPD in attenuating ARDS in COVID-19. The pathology results, based on four trials (*n* = 315), suggest a significant improvement in C-reactive protein levels in patients treated with QFPD and pharmaceutical drugs (*p* < 0.0001 compared with pharmaceutical drugs alone), although no significant change was found in white blood cell counts. 

Most of the RCTs on QFPD were sourced from China, with few studies published in English databases. An English clinical retrospective study with 63 patients with confirmed COVID-19 found that a 14-day treatment of QFPD (two consecutive courses, each course lasting 3 days, without a pause between the courses), when used together with pharmaceutical drugs (selected from interferon, lopinavir or arbidol), demonstrated a similar curative effect (CT and symptoms scores, mortality rates) over the same length of hospitalization compared to pharmaceutical treatment alone. However, the QFPD group showed a greater anti-inflammatory effect, particularly anti-pulmonary inflammation as evidenced by reduced C-reactive protein, total lymphocyte count and lactate dehydrogenase (LDH). These results highlight the therapeutic value of QFPD in attenuating the severity of ARDS in COVID-19 through its prominent anti-inflammatory activity [91]. 

Two in vitro studies also showed the ability of QFPD in reducing IL-6, TNF-α and CXCL-10 under the stimulation of SARS-CoV-2 or LPS [92,93]. It is worth mentioning that the anti-inflammatory action of QFPD was shown to be associated with the inhibition of phosphorylation of IκBα and the activation of the NF-κB signalling pathway [92].

#### 3.2.5. Xuanfei Baidu Decoction 

Xuanfei Baidu (XFBD) was developed by Academician Boli Zhang and Professor Qingquan Liu and is listed in the “three medicines and three prescriptions” of TCM for the treatment of COVID-19 patients. It is a large formulation that consisted of 13 herbal ingredients including *Prunus sibirica* L. (Kuxing ren), *Verbena officinalis* L. (Mabiancao) and *Ephedra sinica* Stapf (Mahuang). 

A pilot randomized clinical trial examined the safety and efficacy of a 1-week treatment of XFBD for forty-two patients with COVID-19. The cohort was randomly divided into XBD (1 pouch of 200 mL each time, 2 times/day) plus conventional medicine (the treatment measures recommended by the “COVID-19 Prevention and Control Program” (Trial), *n* = 22) and conventional medicine alone (*n* = 20). The combined treatment showed significantly reduced clinical symptoms than that of the conventional medicine group including cough, fever and fatigue. The combined treatment also suppressed the elevated number of white blood cells and lymphocytes to normal parameters, suggesting the potential capacity of XFBD in preventing systemic inflammation [94]. Later, another randomized clinical trial confirmed the antiviral activity of XFBD in 103 patients with ordinary-type COVID-19, with the group using combined XFBD and conventional treatment showing the greatest outcome, followed by the Ganlu Xiaodu decoction with conventional treatment, and then conventional treatment only. However, the anti-inflammatory activity was not studied in this trial [95]. 

The anti-inflammatory mechanisms of XFBD were studied in ALI animal models. Zhou et al. (2023) revealed that the action of XFBD was relevant to the regulation of the neutrophils-mediated immune responses, including neutrophil extracellular traps (NETs) formation and the generation of platelet-neutrophil aggregates in LPS-induced ALI mice. The regulatory effect on NETs formation was mediated via the C-X-C Motif Chemokine Ligand 2 (CXCL2)/C-X-C Chemokine Receptor Type 2 (CXCR2) axis [96]. The action of XFBD in suppressing activated macrophages and neutrophils were further investigated in the study from Wang et al. (2022). Their results demonstrated that XFBD reduced macrophages and neutrophils infiltration in LPS-induced ALI and thus improved pulmonary injury [97]. 

At the molecular level, XFBD acted on immunoglobulin G immune complex (IgG-IC)-triggered inflammatory responses in macrophages by inhibiting JAK2/STAT3/SOCS3 and NF-κB signalling pathways [98]. The inhibitory effects of XFBD on the NF-κB signalling pathways were confirmed by the study from Zhu et al. (2023) [99]. In their study, XFBD inhibited proinflammatory factor levels and NF-κB signalling in LPS-induced RAW264.7 cells. The same findings were shown in LPS-induced ALI mouse models. A review published in 2023 summarised the detailed anti-inflammatory activities of XFBD and its phytochemicals which may contribute to the action including ephedrannin B, glycyrrhizin and quinoline-2-carboxylic acids [100]. However, the ability of XFBD to prevent or treat CS has not been thoroughly studied and the mechanisms remain to be explored.

#### 3.2.6. Reduning Injection 

Reduning injection (RDN) is used for upper respiratory tract infection and acute bronchitis. The key ingredients of RDN include *Lonicera japonica* Thunb. (Jinyinhua), *Artemisia annua* L. (Qinghao) and *Gardenia jasminoides* Ellis (Zhizi). 

Wang et al. found that RDN (Jiangsu Kanion Pharmaceutical Co., Ltd., Nanjing, China, Lot No. 160807) treatment (16 or 8 mL/kg/day) significantly reduced the mortality and increased the survival rate in both LPS and CLP-induced septic mice. The pharmacological action of RDN was associated with reduced circulatory inflammatory cytokines including TNF-α, IL-6 and IL-10, possibly through inhibition of the HMGB1/TLR4/NF-κB/MAPKs signalling pathways [101], alleviating the damage in the lungs, liver and kidneys. 

#### 3.2.7. Other Promising Chinese Herbal Medicines or Active Ingredients

- Curcumin

Curcumin is an orange–yellow polyphenol derived from the Curcuma longa L. and has broad anti-inflammatory and anti-cytokine activities [102,103]. Mounting evidence is available to support the use of curcumin for viral pneumonia and ARDS [104]. 

Curcumin was found to improve survival and regulate proinflammatory cytokines (TNF-α, IL-6 and IL-1β) and anti-inflammatory cytokines (IL-2 and IL-10) in mice with sepsis/ALI induced by CLP [103]. Curcumin also significantly decreases the levels of TNF-α and IL-6 in mice with CLP-induced polymicrobial abdominal sepsis [103,105]. In line with this, curcumin dramatically diminished IL-1β, IL-6 and TNF-α productions in the BAL cells [104]. 

Curcumin has broad anti-cytokine activity and has been found to decrease MCP1, MIPI1, GROα, GROβ, IP10, SDF1, MMP-2, IFN-γ and MMP-9. These cytokines regulate the activity of immune cells and inflammatory response and promote fibrosis in the lung after infection [106,107]. 

Most studies agreed that the mode of action of curcumin underpinning its anti-inflammation activity is very versatile, involving NF-κB, COX-2, Nrf2, PPAR-γ and JNKP38 signalling pathways. A study by Yin et al. showed that curcumin has a direct inhibitory effect on NLRP3 inflammasome activation in macrophages from high-fat diet-induced insulin resistance in wide-type C57BL/6 mice, and NLRP3 deficiency abrogated curcumin inhibition of IL-1b and IL-18 production suggesting that the inhibitory effect of curcumin in SIRS may be related to its action in NLRP3 inflammasome [102]. Further study is warranted to examine the effect of curcumin in reversing CS via the action on NLRP3 in SARS-CoV-2 infection. 

Curcumin’s poor solubility and bioavailability has limited its clinical use. Li et al. developed a bio-nano system to improve the targeting ability and bioavailability of curcumin. The results showed that the delivery system significantly decreased the levels of TNF-α and IL-6 in mice with ALI induced by LPS compared to curcumin in its original form [108].

- Babaodan

Babaodan (BBD), a Chinese herbal formula, consists of Bovis Calculus (Niuhuang huang), Fel Serpentis (She dan), Cornu Saigae Tataricae (Lingyangjiao), Margarita (Zhenzhu), Moschus (Shexiang) and Notogiseng Radix et Rhizoma (Sanqi). BBD has been used clinically for the treatment of various infectious diseases, including viral hepatitis and non-infectious liver injury [109,110,111,112]. In a post-viral bacterial infection-induced pneumonia mouse model, BBD increased the survival rate which was associated with significantly reduced serum IL-6 levels. In additional, BBD (3 capsules, by mouth, twice a day, 1.8 g/day for 14 days) was administered to seven severely ill COVID-19 patients as a supplementary therapy to antiviral agents and methylprednisolone. The results showed, via chest CT scan imaging, that all patients recovered from ARDS and none of them progressed to a critically ill status [113]. Their study indicated that BBD controls excessive immune responses which may thus represent a cytokine-targeted agent that could be considered to treat COVID-19-induced ARDS.

- Qiang-xin 1 formula

Qiang-Xin 1 (QX1) is a traditional Chinese herbal formula developed by Prof. Lijuan Huang, a nationally celebrated traditional Chinese medicine expert [114]. It has been used for the treatment of heart failure for more than 30 years [114]. The main herbal ingredients in the QX1 formula consist of Shui hong hua zi (*Polygonum orientale* L.), 30 g; Huang qi [*Astragalus membranaceus* (Fisch.) Bge. var. *mongholicus* (Bge.) Hsiao], 30 g; Fu ling [*Poria cocos* (Schw.) Wolf), 20 g; Dan shen (*Salvia miltiorrhiza* Bge.), 20 g; and Wu wei zi (*Schisandra chinensis* (Turcz.) Baill.], 10 g. In a CLP surgery-induced sepsis model of mice, the administration of 5–20 g/kg of the QX1 decoction reduced sepsis-induced upregulated levels of serum IFN-γ, IL-1β, IL-3, IL-6, IL-17, IL-4, IL-10 and TNF-α. The actions were associated with the downregulation of calcium/calmodulin-dependent protein kinase II (CaMKII), MAPK (P38, ERK1/2, and JNK) and TLR4/NF-κB signalling pathways and upregulation of the PI3K/AKT pathway [115].

- Cardamonin

Cardamonin (20,40-dihydroxy-60-methoxychalcone, CDN) is a chalcone found mainly in the seeds of Alpinia katsumadai (Caodoukou in Chinese), a medicinal herb that has been widely used to treat digestive system-related diseases for years. CDN has shown considerable anti-inflammatory, anti-cancer, anti-oxidative and vasorelaxant activities [116,117,118,119,120]. In an LPS-induced septic shock mouse model, CDN ameliorated septic shock and IL-1β production and improved the survival of mice suffering from septic shock. In primary bone-marrow-derived macrophages, PBMCs and human peripheral blood mononuclear cells, CDN suppressed caspase-1 activation, TNF and IL-1β secretion and inhibited NLRP3 inflammasome activation [121]. The broad-spectrum and specific inhibitory effects of CDN on NLRP3 inflammasome make it an attractive therapeutic agent in CS-induced COVID-19 ARDS.

- Yam Glycoprotein

Yam glycoprotein, separated from traditional Chinese yam, has shown anti-inflammatory and immunomodulatory effects. In lipopolysaccharide (LPS)-induced ALI mouse models, glycoprotein markedly depressed LPS-induced lung wet/dry ratios, myeloperoxidase activity, malondialdehyde content, superoxide dismutase and glutathione peroxidase depletion and the contents of inflammatory cytokines IL-1β, IL-6 and TNF-α. The mechanisms were associated with the inhibition of TLR4/NF-κBp65 signalling activation and NLRP3 inflammasome. As such, glycoprotein may offer some therapeutic value as an NLRP-3 inflammasome silencer for ARDS [122]. 

Please see Table 1 for the action of herbal formulations that have potential to suppress pro-inflammatory cytokines and associated mechanisms under CS in relation to ARDS.

## 4. Discussion

In this paper, the pathological mechanisms of CS-induced COVID-19 ARDS are reviewed. Although the pathogenesis is very complex and involves a cascade of signalling and molecular events, the knowledge on key mediators and turning points is becoming clearer. Thus far, the scientific literature is lacking in terms of specific information for pro-inflammatory and anti-inflammatory production in COVID-19 ARDS. This includes which pro-inflammatory cytokines remain elevated and the time that they remain elevated for, to assist in confirming the stage of the disease. This knowledge may be essential for the status and severity of the SARS-CoV-2 infection and can contribute to the development of effective treatment strategies.

Since it has been shown that COVID-19 ARDS is a life-threating event and may bring a plethora of complications, its prevention should be the fundamental principle at an early stage to postpone the onset of complications or even prevent it from occurring. While vaccines are widely distributed worldwide for disease control and prevention, the population who are not suitable for vaccines or who are at higher risk, including those with comorbidities or undergoing immunosuppressive treatment, require effective treatment to minimise the complications of COVID-19. Studies have shown that people that have a transplant, are undergoing chemotherapy or radiation therapy or are immune-compromised are prone to develop CS [125]. Thus, COVID-19 patients with high-risk factors should be carefully monitored to prevent the occurring of CS. Many cytokines and signalling pathways are known to play a role in the pathological mechanisms of both local acute inflammation, chronic inflammation and CS. However, the exact contribution of local or chronic inflammation to CS and the pathological role of chronic inflammation in COVID-19 ARDS remains unclear. Several studies have suggested that mental health issues such as stress and insomnia are the underlying risk factors of systemic inflammation [126,127]. However, whether they also contribute to the onset of CS in ARDS warrants exploration. 

Many single cytokines and associated signalling pathways are shown to be involved in the escalation of the inflammatory cascade and contribute to the development of CS. Based on this knowledge, therapeutic targets are discussed to block the major cytokines with the purpose of preventing the further development of or attenuating CS. However, the single-compound, single-target approach has not been able to achieve consistently satisfactory clinical outcomes, especially when compared to the side effects of the treatment. Several studies have demonstrated that addressing two or more cytokines may bring desired outcomes compared to a single cytokine. However, the rationale is not clear, and more studies are warranted to evaluate the best combination of cytokines as therapeutic targets to effectively prevent or delay the progression of CS. 

Chinese herbal medicines have shown beneficial effects in attenuating CS and reducing the mortality of COVID-19 ARDS in clinical trials. Chinese herbal formulations that showed relatively strong clinical evidence as an adjunct therapy or individual treatment for COVID-19 ARDS include Xuebijing Injection, Huashi Baidu Formula, Lianhua Qingwen granules/capsules, Xuanfei Baidu decoction and Qingfei Paidu Decoction. However, the majority of these trials were conducted in China. Since COVID-19 is a global pandemic, it is essential to evaluate these herbal interventions in a diverse population. Additionally, multiple methodological issues were identified. Most studies did not conduct blinding and/or randomisation, and the interventions in these studies were not clearly defined. Furthermore, the measurement outcomes mainly focused on viral load and COVID-19-related symptoms (i.e., cough, fever, lung injury), leading to a lack of data on CS-related biomarkers. Clinical trials with rigorous designs involving diverse ethnicities and high-risk populations are needed to further evaluate the efficacy and safety of Chinese herbal medicine, and more CS-related outcome measurements should be included. 

The complex chemical composition of these formulations poses a significant challenge for elucidating their mechanisms, and in particular, which component(s) is/are responsible for the therapeutic effect. Most of the herbal formulations consist of more than five herbal ingredients and standardisation of these interventions is generally lacking, which makes it hard to validate/replicate these studies. More research is also needed to understand the interactions between herbal medicines and conventional drugs, which are yet to be described and urgently needed [128]. In the aforementioned RCTs, Chinese herbal medicine was largely given alongside antiviral medication and there exists the possibility for pharmacodynamics and pharmacokinetic herb–drug interactions [129]. In addition, it is not yet understood why the combination of Chinese herbal medicine and conventional therapy improves clinical outcomes, which would require further investigation to elucidate the mechanism of these effects. Thus far, there is a lack of high-quality data on the safety of combining Chinese herbal and conventional medicine for the treatment of CS, which could also be mitigated through the development of larger, rigorous RCTs in diverse populations.

The results of the existing in vitro and in vivo studies have demonstrated the therapeutic potential of several promising bioactive molecules from herbal medicine. Among them, curcumin appears to be the most promising single bioactive compound for CS through a multi-targeted mode of action. However, the therapeutic effect of curcumin in human trials is yet to be determined, partly due to its low bioavailability. More research is warranted using new curcumin preparations with an enhanced bioavailability profile in CS-induced COVID-19 ARDS.

## 5. Conclusions 

The pathological mechanisms of CS in COVID-19 ARDS involve a complex cascade of the activation of signalling and pro-inflammatory cytokines. Multiple therapeutic targets have been identified. Early evidence exists to demonstrate the therapeutic value of Chinese herbal medicine as an adjunct therapy in improving clinical symptoms and reducing the mortality of patients with COVID-19 ARDS. Further research is warranted to determine the efficacy and mechanisms of action of Chinese herbal medicine for CS-associated COVID-19 ARDS. 

## Figures and Tables

**Table 1:** The action of herbal formulations that have potential to suppress pro-inflammatory cytokines and associated mechanisms under CS in relation to ARDS.

Chinese Herbal Medicine	Cytokines	Molecular Mechanism	References
XBJ	IL-1β, IL-6, IL-8, IL-10, TNF-α, IL-2, IL-4	Involves the inhibition of MAPK, NF-κB and other signalling pathways	[72,74,75,78]
HSBD	IL-6	Involves the inhibition of MAPK, NF-κB and STAT1 signalling	[80,123]
LHQW	IL-6, IP-10, MCP-1	The IL-6 receptorIL-6/IL-6 receptor subunit beta were the main targets, but signalling pathways were not investigated	[87,88,89]
QFPD	CXCL-10, IL-6, TNF-α	Inhibition of IκBα phosphorylation and NF-κB signalling pathways	[92,93]
QFBD	IL-6, TNF-α, IL-1β, IL-10, MCP-1, IL-18	Inhibited CXCL2/CXCR2, JAK2/STAT3/SOCS3, PD-1/IL17A and NF-κB signalling pathways	[96,97,98,99,100]
RDN	IL-6, IL-10, TNF-α	Postulated to be through inhibition of HMGB1/TLR4/NF-κB/MAPKs signalling pathways	[101]
Curcumin	IL-1β, IL-6, MCP-1, TNF-α	Multifactorial mechanism involving NF-κB, COX-2, Nrf2, PPAR-γ and JNKP38 signalling pathways	[103,104,105,106,107]
BBD	IL-6	Postulated to act through modulation of the MAPK and NF-κB signalling pathways	[113,124]
QX1	IL-1β, IL-3, IL-4, IL-6, IL-10, IL-17, TNF-α	Multifactorial mechanism involving CaMKII, MAPK, NF-κB, PI3K/AKT signalling pathways	[115]
CDN	IL-1β, TNF-α	Inhibition of the NLRP3 inflammasome	[121]
Yam Glycoprotein	IL-1β, IL-6, TNF-α	Inhibition of the NLRP3 inflammasome and NF-κB signalling	[122]

## Data Availability

Not applicable.
